# Lymphocyte-specific protein tyrosine kinase (Lck) interacts with CR6-interacting factor 1 (CRIF1) in mitochondria to repress oxidative phosphorylation

**DOI:** 10.1186/s12885-015-1520-6

**Published:** 2015-07-26

**Authors:** Shahrooz Vahedi, Fu-Yu Chueh, Bala Chandran, Chao-Lan Yu

**Affiliations:** 1 Department of Microbiology and Immunology, H. M. Bligh Cancer Research Laboratories, Chicago Medical School, Rosalind Franklin University of Medicine and Science, 3333 Green Bay Road, North Chicago, IL 60064 USA; 2 Department of Biomedical Sciences, College of Medicine, Chang Gung University, 259 Wenhua 1st Road, Taoyuan City, 33302 Taiwan Republic of China

**Keywords:** Lck, Leukemia, Mitochondria, Oxidative phosphorylation, Electron transport chain, Cancer metabolism, Mitoribosome, CRIF1, Tid1, Proximity ligation assay

## Abstract

**Background:**

Many cancer cells exhibit reduced mitochondrial respiration as part of metabolic reprogramming to support tumor growth. Mitochondrial localization of several protein tyrosine kinases is linked to this characteristic metabolic shift in solid tumors, but remains largely unknown in blood cancer. Lymphocyte-specific protein tyrosine kinase (Lck) is a key T-cell kinase and widely implicated in blood malignancies. The purpose of our study is to determine whether and how Lck contributes to metabolic shift in T-cell leukemia through mitochondrial localization.

**Methods:**

We compared the human leukemic T-cell line Jurkat with its Lck-deficient derivative Jcam cell line. Differences in mitochondrial respiration were measured by the levels of mitochondrial membrane potential, oxygen consumption, and mitochondrial superoxide. Detailed mitochondrial structure was visualized by transmission electron microscopy. Lck localization was evaluated by subcellular fractionation and confocal microscopy. Proteomic analysis was performed to identify proteins co-precipitated with Lck in leukemic T-cells. Protein interaction was validated by biochemical co-precipitation and confocal microscopy, followed by *in situ* proximity ligation assay microscopy to confirm close-range (<16 nm) interaction.

**Results:**

Jurkat cells have abnormal mitochondrial structure and reduced levels of mitochondrial respiration, which is associated with the presence of mitochondrial Lck and lower levels of mitochondrion-encoded electron transport chain proteins. Proteomics identified CR6-interacting factor 1 (CRIF1) as the novel Lck-interacting protein. Lck association with CRIF1 in Jurkat mitochondria was confirmed biochemically and by microscopy, but did not lead to CRIF1 tyrosine phosphorylation. Consistent with the role of CRIF1 in functional mitoribosome, shRNA-mediated silencing of CRIF1 in Jcam resulted in mitochondrial dysfunction similar to that observed in Jurkat. Reduced interaction between CRIF1 and Tid1, another key component of intramitochondrial translational machinery, in Jurkat further supports the role of mitochondrial Lck as a negative regulator of CRIF1 through competitive binding.

**Conclusions:**

This is the first report demonstrating the role of mitochondrial Lck in metabolic reprogramming of leukemic cells. Mechanistically, it is distinct from other reported mitochondrial protein tyrosine kinases. In a kinase-independent manner, mitochondrial Lck interferes with mitochondrial translational machinery through competitive binding to CRIF1. These findings may reveal novel approaches in cancer therapy by targeting cancer cell metabolism.

**Electronic supplementary material:**

The online version of this article (doi:10.1186/s12885-015-1520-6) contains supplementary material, which is available to authorized users.

## Background

Mitochondria are double-membrane cellular organelles responsible for producing the majority of energy essential for cell survival. In addition to regulating energy metabolism by oxidative phosphorylation (OXPHOS) of glucose and fatty acids, mitochondria are known to control cell migration, apoptosis, and intracellular signaling [1]. A growing body of literature indicates the importance of mitochondrial health and activity at different stages of cancer development [2]. So far, many genetic and non-genetic abnormalities have been linked to mitochondrial dysfunction [3]. These abnormalities are most often caused by deregulation of mitochondrial proteins that make up the electron transport chain (ETC) complex, which is a major player in mitochondrial respiration [4]. Cancer cells can be metabolically distinct from normal cells and rely more on aerobic glycolysis than mitochondrial OXPHOS. This shift in energy metabolism allows more resources to be converted into biomass for cancer cell’s uncontrolled growth and proliferation [5]. On the other hand, accumulating reports also suggest that cancer cells may maintain oxidative metabolism under normoxic conditions [6–8]. The tumor microenvironment, such as stromal fibroblasts, can play an active role is modulating cancer cell’s metabolism as well [9].

Mitochondrial respiration is tightly regulated by the ETC complex embedded in the mitochondrial inner membrane [10]. The majority of ETC proteins are encoded by the nuclear genome, translated in the cytoplasm, and then translocated to mitochondria. However, thirteen ETC proteins are encoded by the mitochondrial circular DNA. In addition to mRNAs for the thirteen polypeptides, the mitochondrial genome encodes additional rRNAs and tRNAs essential for the assembly of mitochondrion-specific translational machinery [11]. Intramitochondrial protein synthesis is carried out by the mitoribosome, a non-canonical ribosome within mitochondrial matrix. Additional proteins encoded by the nuclear genome, such as CR6-interacting factor 1 (CRIF1) and tumorous imaginal disc 1 (Tid1), are also components of mitoribosome and participate in subsequent insertion of properly folded proteins into the inner membrane to assemble functional ETC complex [12]. Because mitochondrion-encoded proteins are key ETC components, mitochondrial OXPHOS can be modulated by the levels of transcription of mitochondrial genomes, its translation, and subsequent post-translational modifications [13–15]. The activity of mitochondrial proteins, including ETC proteins, can be regulated by reversible phosphorylation [16]. In addition to serine and threonine phosphorylation, the importance of tyrosine phosphorylation has been demonstrated by recent studies on mitochondrial proteins. Numerous kinases and phosphatases are known to translocate to the mitochondria and dynamically change the phosphorylation status and activity of mitochondrial proteins.

Many protein tyrosine kinases (PTKs) are traditionally known as signal transducers in transmitting signals to the nucleus and mitochondria [17]. They are important in modulating nuclear and mitochondrial activity, which in turn regulate diverse cellular functions in response to extracellular stimuli. Recent findings further demonstrate that PTKs can translocate to the mitochondria and directly participate in regulating mitochondrial activity. Several receptor PTKs, including epidermal growth factor receptor (EGFR), fibroblast growth factor receptor 1 (FGFR1), and ErbB2, have been shown to translocate to mitochondria [18–20]. Tyrosine phosphorylation of cytochrome *c* oxidase subunit II (COII), a mitochondrion-encoded ETC protein, results in reduced ETC activity [18]. Consistent with the characteristic metabolic shift observed in cancer cells, mitochondrial localization of FGFR1 and ErbB2 contributes to reduced OXPHOS in lung and breast cancer, respectively [19, 20]. Similarly, mitochondrial translocation of non-receptor PTKs, such as Src, has been reported [21]. Mitochondrial c-Src and its phosphorylation of substrates are associated with elevated ETC activity and survival of rat brain tissue and human glioblastoma cells [22, 23]. In contrast, as the effector protein downstream of EGFR, mitochondrial c-Src phosphorylates COII and reduces ETC activity [18]. It suggests that mitochondrial c-Src may function differently depending on the cellular context. Mitochondrial localization of other Src family kinases (SFKs), including Fyn, Lyn and Fgr, has also been proposed [24]. Nevertheless, it still remains largely unknown how different SFKs function inside the mitochondria either in normal cells or in cancer cells.

Lymphocyte-specific protein tyrosine kinase (Lck) is a SFK predominantly expressed in T-cells to regulate T-cell development and homeostasis [25, 26]. As a plasma membrane-associated protein, Lck is the key PTK that initiates intracellular signaling from T-cell receptor (TCR) on the surface [27, 28]. Lck gene is localized near the chromosomal region with high frequency of translocation in cancer [29]. Overexpression and aberrant activity of Lck have been reported in both acute and chronic leukemias [30]. In addition to leukemia, abnormal Lck expression is detected in solid tumors, including brain [31], breast [32], colorectal [33], and prostate [34] cancer. In breast cancer, Lck promotes tumor progression and angiogenesis [35]. Involvement of Lck in radiation-induced proliferation and resistance in glioma patients has also been reported [31]. Our earlier studies further demonstrated the oncogenic property of active Lck kinase in both T and non-T cells [36, 37]. Recently, we showed that oncogenic Lck kinase translocated to the nucleus and upregulated the expression of a nuclear target gene important in hematological malignancies [38]. This non-canonical mode of PTK signaling suggests that, like c-Src, Lck may also exhibit additional functions in mitochondria. In this study, we specifically tested this hypothesis in the context of T-cell leukemia and employed proteomics to define the underlying mechanisms. Our results demonstrate that Lck represses oxidative phosphorylation through competitive binding with mitochondrial CRIF1 in a kinase-independent manner.

## Methods

### Cell lines and reagents

The human T-cell line Jurkat clone E6.1 and its Lck-deficient derivative Jcam clone 1.6 were purchased from American Type Culture Collection (ATCC, Manassas, VA, USA). Jurkat E6.1, Jcam 1.6 and the mouse LSTRA leukemia cell lines were maintained as described previously [39]. CRIF1 knock-down stable cell lines were generated in Jcam using lentiviral transduction. CRIF1 shRNA (sc-97804-V) and scrambled shRNA control (sc-108080) lentiviral particles were purchased from Santa Cruz Biotechnology (Dallas, TX, USA). After 24-h starvation, 10^4^ Jcam cells were harvested and resuspended in 50 μl of freshly thawed virus mixture (2 × 10^5^ infectious units of virus). After 6-h incubation, 500 μl of complete RPMI were added. After one day of recovery, puromycin was added to a final concentration of 14 μg/ml to select for stably transduced cells. Efficiency of CRIF1 knock-down was evaluated by Western blot and real-time PCR analyses.

### Subcellular fractionation

Mitochondrial fraction was isolated by hypotonic lysis and differential centrifugation as described previously [39]. Briefly, cells were washed in phosphate-buffered saline (PBS) and then homogenized by passing through a 27-gauge needle in ice-cold hypotonic buffer. Light microscopy was used to ensure cell rupture before proceeding to the next step. Mitochondria-enriched heavy membrane fraction (mitochondrial fraction) was collected by differential centrifugation. Fraction purity was verified by immunoblotting of specific markers.

### Immunoprecipitation and immunoblotting

Whole cell lysates were prepared by solubilizing cell pellets in RIPA buffer [39]. Target proteins were either immunoprecipitated or directly detected from whole cell lysates after SDS-PAGE using specific antibodies according to manufacturers’ instructions. Mitochondrial proteins were extracted from heavy membrane pellets using either high salt buffer for co-immunoprecipitation [40] or 1 % NP-40 lysis buffer for direct immunoblotting. Antibodies specific for Lck and CRIF1 were purchased from Santa Cruz Biotechnology. Antibodies specific for VDAC1 (voltage-dependent anion channel 1), ND1 (NADH dehydrogenase subunit 1), COI (cytochrome *c* oxidase subunit I) and Tid1 were purchased from Abcam (Cambridge, MA, USA). Anti-COIV (cytochrome *c* oxidase subunit IV) antibody was purchased from Bethyl Laboratories (Montgomery, TX, USA). Antibodies specific for phospho-Src family (Tyr416) and GAPDH (glyceraldehyde 3-phosphate dehydrogenase) were purchased from Cell Signaling Technology (Danvers, MA, USA). Anti-phosphotyrosine antibody (clone 4G10) was purchased from EMD Millipore (Billerica, MA, USA). Appropriate secondary antibodies conjugated with horseradish peroxidase were used in enhanced chemiluminescence system to detect signals. Conformation-specific antibodies that do not recognize heavy chains in the immunoprecipitates (from Affymetrix eBioscience, San Diego, CA, USA) were also used to minimize interference in detecting Lck signals. For signal quantitation, the bands were digitalized using the AlphaImager 2200 (ProteinSimple, San Jose, CA, USA) and analyzed by the ImageJ software.

### Confocal immunofluorescence microscopy

Live cells were incubated with 100 nM of MitoTracker Deep Red (Life Technologies, Grand Island, NY, USA) for 20 min under regular culture condition or left unstained as a negative control. Stained cells were washed with PBS, adhered to 10-well slides, fixed, and permeabilized as previously described [40]. Cells were blocked with Image-iT FX signal enhancer (Life Technologies) for 15 min at room temperature, and then either singly or doubly stained with primary antibodies. Subsequent labeling with Alexa Fluor-conjugated secondary antibodies and DAPI counterstain (Life Technologies) were performed to visualize primary antibodies and nuclei, respectively. Stained cells were viewed using the Olympus FV10i fluorescence confocal microscope. Images were analyzed using the Fluoview software (Olympus, Melville, NY, USA).

### *In situ* proximity ligation assay (PLA) microscopy

PLA was performed using the DuoLink PLA Kit (Sigma-Aldrich, St. Louis, MO, USA) to detect close-range protein-protein interactions under a fluorescence microscope according to manufacturer’s protocol. Briefly, 10^4^ cells were seeded on each well of 10-well slides. Adhered cells were fixed with 4 % paraformaldehyde for 15 min at room temperature, and then permeabilized with 0.2 % Triton X-100. After treatment with DuoLink blocking buffer for 30 min at 37 °C, cells were incubated with diluted primary antibodies from two different species for another hour at 37 °C. After washing, cells were incubated with species-specific PLA probes and two additional oligonucleotides under conditions that facilitate hybridization only in close proximity (<16 nm). A ligase was added to join the hybridized oligonucleotides to form a closed circle. A rolling-circle amplification step with polymerase was then performed to generate a concatemeric product extending from the oligonucleotide arm of the PLA probe. The amplified product can be visualized with fluorophore-labeled oligonucleotides after hybridization as distinct fluorescent dots under a fluorescence microscope. For negative controls, samples were treated as described above, except that no primary antibodies were added. Slides were also counterstained with DAPI to visualize the nuclei.

### Quantitative real-time PCR analysis

Total RNAs were extracted by TRIzol (Life Technologies), treated with RQ1 RNase-free DNase (Promega, Madison, WI, USA), and then reverse transcribed using High Capacity cDNA Reverse Transcription Kit (Applied Biosystems, Foster City, CA, USA) into cDNAs. Quantitative real-time PCR using SYBR Green chemistry (Applied Biosystems) was performed according to standard protocol using an annealing temperature of 60 °C for all primer sets. Relative fold values were obtained using ΔΔCT method by normalization to β-actin. Primers for various human genes are itemized below.

- ND1 (forward): 5′-GAGCAGTAGCCCAAACAATCTC-3′
- ND1 (reverse): 5′-AAGGGTGGAGAGGTTAAAGGAG-3′
- COI (forward): 5′-CAATATAAAACCCCCTGCCATA-3′
- COI (reverse): 5′-GCAGCTAGGACTGGGAGAGATA-3′
- COIV (forward): 5′-TGGATGAGAAAGTCGAGTTG-3′
- COIV (reverse): 5′-CTTCTGCCACATGATAACGA-3′
- CRIF1 (forward): 5′-GGTGGTCCCCGGTTCGTTATGG-3′
- CRIF1 (reverse): 5′-CTCGCGCCTCCTTCTTCCGTTTCT-3′
- Actin (forward): 5′-CGCAGAAAACAAGATGAGATTG-3′
- Actin (reverse): 5′-ACCTTCACCGTTCCAGTTTTTA-3′

### Mass spectrometry

Whole cell pellet of LSTRA was solubilized with 1 % NP-40 lysis buffer. Lysates with 500 μg of proteins were immunoprecipitated with 2 μg of anti-Lck antibody or control mouse IgG overnight. Immunoprecipitates were resolved using 4–20 % gradient SDS-PAGE (Bio-Rad, Hercules, CA, USA) and visualized with Coomassie blue staining. A total of eight bands specifically present in the Lck immunoprecipitates, but not in the IgG control, were cut out from the gel. Proteins extracted from gel slices were analyzed by mass spectrometry using liquid chromatography-electrospray ionization-tandem mass spectrometry (LC-ESI-MS/MS) based approach at the Midwest Proteome Center, Rosalind Franklin University of Medicine and Sciences (RFUMS).

### Electron microscopy

Jurkat and Jcam cells were washed with warm PBS and prefixed in 0.2 % paraformaldehyde and 0.25 % glutaraldehyde for 15 min at room temperature. Prefixed cells were centrifuged and resuspended in ice-cold fixation solution (2 % paraformaldehyde and 2.5 % glutaraldehyde) overnight. Cell pellets were washed in 0.1 M Sorensen’s sodium phosphate buffer (SPB), pH 7.4 at room temperature for 15 min, followed by post-fixation with 1 % OsO4 and 1.5 % K4Fe(CN) in SPB for 1 h. After washing, cell pellets were dehydrated through an ascending ethanol series and embedded in Epon 812 resin. Ultra-thin sections were cut with a diamond knife and Leica UC-6 ultramicrotome, and collected onto 200-mesh grids. Sections on grids were contrasted using Reynolds’ lead citrate stain and then viewed using a JEOL JEM-1230 transmission electron microscope (Peabody, MA, USA). Digital images were collected using a Hamamatsu Orca high resolution CCD camera.

### Oxygen consumption analysis

Oxygen consumption rate was measured using a Clark-type electrode equipped with the 782 oxygen meter (Strathkelvin Instrument, North Lanarkshire, Scotland) with a water circulation system to maintain the reaction condition at 37 °C. Cells were washed with warm PBS and then adjusted to a final concentration of 10^7^ cells per ml in TD assay buffer (0.137 M NaCl, 5 mM KCl, 0.7 mM Na_2_HPO_4_, 25 mM Tris, pH 7.4) [41]. Five million cells were transferred to water-jacked chamber MT-200 (Strathkelvin Instrument) to record their oxygen consumption rate. Homogenous distribution of cells was maintained throughout the recording process by constant magnetic stirring. Other than the measurement of basal oxygen consumption rates, oligomycin (Cayman Chemical, Ann Arbor, MI, USA) was also added to the same chamber at a final concentration of 500 nM to determine the oxygen consumption rates independent of ATP. Data were analyzed using the SI 782 Oxygen System software version 3.0 (Warner Instruments LLC, Hamden, CT, USA) and normalized to cell number.

### Mitochondrial superoxide measurement

Mitochondrial superoxide was measured using MitoSOX Red (Life Technologies) according to manufacturer’s protocol. MitoSOX Red is a fluorescent dye that targets mitochondria in live cells and is specifically oxidized by superoxide. Approximately 10^6^ cells were stained with 1 μM MitoSOX Red for 20 min at 37 °C, washed in warm PBS and then analyzed by the LSR II flow cytometer (BD Bioscience, San Jose, CA, USA). Mean fluorescence intensity from oxidized MitoSOX (ex/em 510/580) positively correlates with mitochondrial superoxide levels.

### Mitochondrial membrane potential measurement

Mitochondrial membrane potential was measured using tetramethylrhodamine, ethyl ester (TMRE) according to manufacturer’s protocol. Mitochondrial membrane potential drives the accumulation of TMRE, a fluorescent dye, within the inner membrane region. Approximately 10^6^ cells were harvested and washed with warm plain RPMI media and then resuspended in TMRE solution (Life Technologies) at the final concentration of 25 nM. After incubation at 37 °C for 30 min, cells were washed and then analyzed by flow cytometry. Mean fluorescence intensity (ex/em 549/575) positively correlates with mitochondrial membrane potential.

### Statistical analysis

Data are presented as mean ± S.E. from at least three independent experiments. The significance of differences was analyzed by Student’s *t*-test (SigmaPlot 11, Chicago, IL, USA). Differences were considered significant when *p* < 0.05.

## Results

### Mitochondrial Lck correlates with mitochondrial dysfunction in leukemia cells

**Fig. 1:**
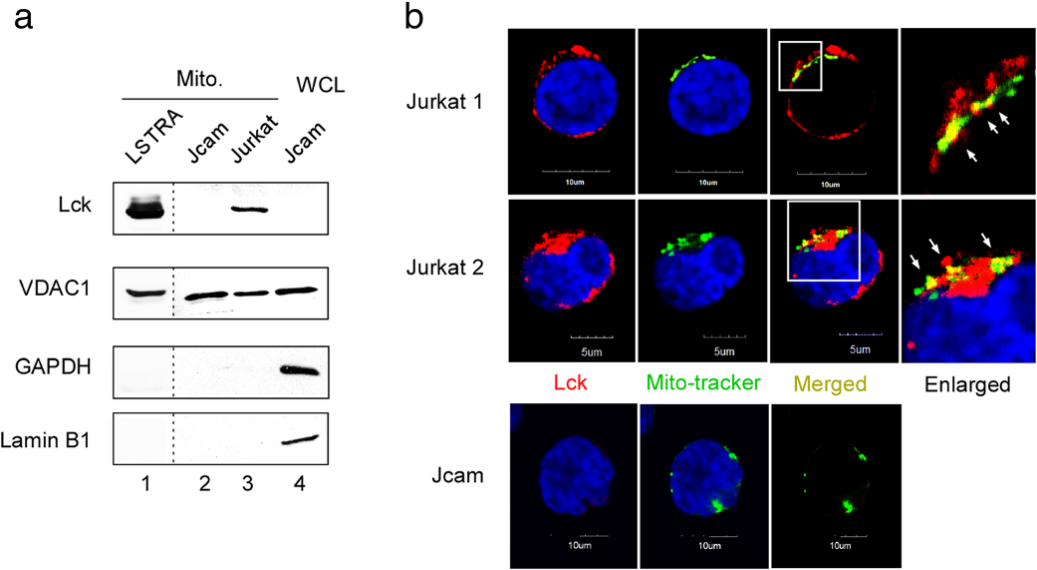
Mitochondrial localization of endogenous Lck protein in both mouse and human leukemia cell lines. (**a**) Mitochondrial (Mito) fractions isolated from three leukemia cell lines were analyzed by Lck immunoblotting. Immunoblotting for VDAC1 (mitochondrial marker), GAPDH (cytoplasmic marker), and lamin B1 (nuclear marker) was performed to verify purity of mitochondrial fractions. Jcam whole cell lysate (WCL) was used as the positive control for markers. LSTRA lysates were analyzed on a separate membrane as shown by the dotted lines. (**b**) Confocal microscopy of three-color fluorescence staining of Jurkat (top and middle panels) and Jcam (bottom panels) cells. An area of Jurkat microscopy (bordered with white lines) is enlarged and shown on the right. Lck (red) and mito-tracker (green) co-localization are shown as yellow dots and depicted by white arrows in the enlarged image. Nuclei are visualized with DAPI staining (blue). Scale bars are shown in the bottom

We reported previously that exogenously expressed oncogenic Lck kinase translocated to the nucleus and activated gene expression through binding to specific promoters [38]. The mouse leukemic T-cell line LSTRA overexpresses Lck kinase and mimics the aggressive form of human large granular lymphocytic leukemia [42]. Similarly, endogenous Lck localizes in the nucleus and activates nuclear gene expression in LSTRA leukemia [38]. To determine whether Lck also translocates to the mitochondrial compartment of LSTRA leukemia, we preformed subcellular fractionation to isolate the mitochondrial fraction. Immunoblotting confirms the presence of mitochondrial Lck in LSTRA cells (Fig. 1a, lane 1). The absence of GAPDH and lamin B1 in the mitochondrial fraction rules out the possibility of contamination from the cytosolic and nuclear compartments, respectively.

Because Lck is overexpressed in LSTRA leukemia, it’s important to determine whether endogenous Lck expressed at normal level also translocates to mitochondria. Therefore, we examined Jurkat, a well-known human leukemic T-cell line, and its derivative, Jcam cell line. Jcam is characterized as an Lck-low Jurkat due to both truncation of Lck that inactivates its kinase activity and its expression at a very low level [43]. Similar to LSTRA, Jurkat cells also have detectable Lck localization in the mitochondria as shown by subcellular fractionation (Fig. 1a, lane 3). Mitochondrial localization of Lck was further validated by confocal microscopy after immunofluorescence staining. As shown in Fig. 1b, Lck and mito-tracker co-localize in Jurkat cells (upper-right panels). Consistent with Lck deficiency, Lck was not detected in Jcam cells either by Western blot (Fig. 1a, top panel) or by immunofluorescence microscopy (Fig. 1b, bottom-left panel).

**Fig. 2:**
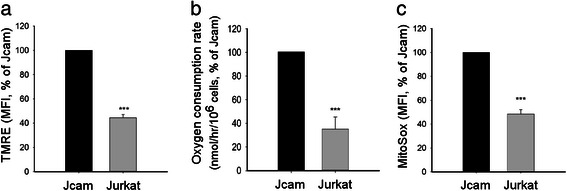
Association between Lck expression and decreased mitochondrial activity. Jurkat and Jcam cells were analyzed for mitochondrial membrane potentials (panel **a**), oxygen consumption rate (panel **b**), and mitochondrial superoxide level (panel **c**). Experimental details are described in Methods. Membrane potentials and mitochondrial superoxide levels are shown as mean fluorescence intensity (MFI) by flow cytometry. Data are presented as percentage of activity in Jurkat as compared to Jcam. Statistical analyses were perform on three independent experiments, ***p < 0.001

Mitochondrial localization of PTKs have been shown to either decrease [20, 18] or increase [23, 22] mitochondrial OXPHOS. In order to determine the effects of Lck on mitochondrial respiration, we compared different mitochondrial functions between Jurkat and Jcam cells. Proper ETC activity creates an electrochemical proton gradient across the mitochondrial inner membrane. The mitochondrial membrane potential (∆Ψm) is an important indicator of mitochondrial health and activity [44]. As shown in Fig. 2a, mitochondrial membrane potential is reduced in Jurkat as compared to Jcam. Oxygen consumption is another important parameter of OXPHOS in evaluating mitochondrial respiration. Consistent with lower ∆Ψm, Jurkat also consumes less oxygen in comparison to Jcam (Fig. 2b). These data are consistent with a previous report indicating concomitant reduction of mitochondrial membrane potential and oxygen consumption mediated by mitochondrial ErbB2 in breast cancer cells [20]. We also measured oxygen consumption rates in the presence of oligomycin, an ATP synthase inhibitor [45]. Our results showed that both basal and ATP-linked oxygen consumption are lower in Jurkat as compared to Jcam cells (see Additional file 1), supporting a decrease of OXPHOS activity in Jurkat cells.

A change in ETC activity is known to alter the levels of mitochondrial reactive oxygen species (ROS) [46]. Therefore, we also examined the levels of mitochondrial superoxide, the precursor of many ROS. As shown in Fig. 2c, there is a similar drop of mitochondrial superoxide levels in Jurkat as compared to Jcam. Taken together, these results demonstrate the presence of endogenous Lck kinase in the mitochondria of both human and mouse leukemic T-cells. The functional data from Jurkat and Jcam comparison also suggest a link between Lck expression and decreased mitochondrial respiration and OXPHOS.

### Lck interacts with CRIF1 in the mitochondria

In order to gain mechanistic insight of how Lck regulates mitochondrial activity, we decided to identify potential Lck-interacting protein(s) by proteomics. Lck immunoprecipitation was performed in LSTRA leukemia to maximize the detection of Lck-associated proteins. After mass spectrometry, data were analyzed based on percent coverage, subcellular locations, and functions. Top hits from our proteomic analysis are summarized in Table 1. Identification of previously known Lck-interacting partners, such as TCR, validates the accuracy of our proteomic approach and analysis. Among other candidates, CRIF1 is of particular interests because of its unique functions in both nuclear and mitochondrial compartments [47–49]. In mitochondria, CRIF1 is involved in the translation of mitochondrion-encoded mRNAs and subsequent insertion of newly synthesized proteins into the inner membrane to form functional ETC complex [12, 50].

**Table 1:** Summary of mass spectrometric analysis of Lck immunoprecipitates from LSTRA lysate

Protein name	Accession number	Symbol	Location	Mass	PEAKS (Score %)	Coverage (%)
**CR6-interacting factor 1**	**269315840**	**CRIF1**	**Mitochondria, Nucleus**	**22 KDa**	**60.6**	**3.15**
ADP/ATP translocase 2	22094075	ANT2	Mitochondria	33 KDa	97.40	28.86
*ADP/ATP translocase 1*	*148747424*	*ANT1*	*Mitochondria*	*33 KDa*	*90.00*	*20.47*
ADP/ATP translocase 4	254692892	ANT4	Mitochondria	35 KDa	51.30	7.81
*T-cell receptor alpha chain V region*	*91435*	*TCR*	*Cell membrane*	*12 KDa*	*6.4*	*8.18*
E3 Ubiquitin-protein ligase	20987377	RNF43	Cytosolic	81 KDa	24	0.99
E3 ubiquitin-protein ligase RNF181	47059206	RNF181	Cytosolic	17 KDa	13.9	5.93
Hermansky-Pudlak syndrome 6 protein homolog	254939591	HPS6	Cytosolic	127 KDa	85.4	4.47
B-cell stimulating factor 3	9910314	BSF-3	Secreted	25 KDa	60.6	3.11
Heat shock protein 90	40556608	HSP90AB1	Cytosolic	83 KDa	99.0	14.36
Glyceraldehyde-3-phosphoate dehydrogenase	6679937	GAPDH	Cytosolic	35 KDa	64.6	9.09
*Lactate dehydrogenase A*	*6754524*	*LDH-A*	*Cytosolic*	*36 KDa*	*64.6*	*9.09*
Transcription initiation factor TFIID subunit 12	12841601	TAF12	Nucleus	17 KDa	99	14.36
Nucleolin	148708273	NCL	Nucleolus	76 KDa	60.6	3.11
GTP-binding protein 2	240120093	GTPBP2	Cytoplasmic, nucleolus	65 KDa	15.3	1.1

Proteins that are the focus of the current study or discussed in the text are indicated as bold and italics, respectively

**Fig. 3:**
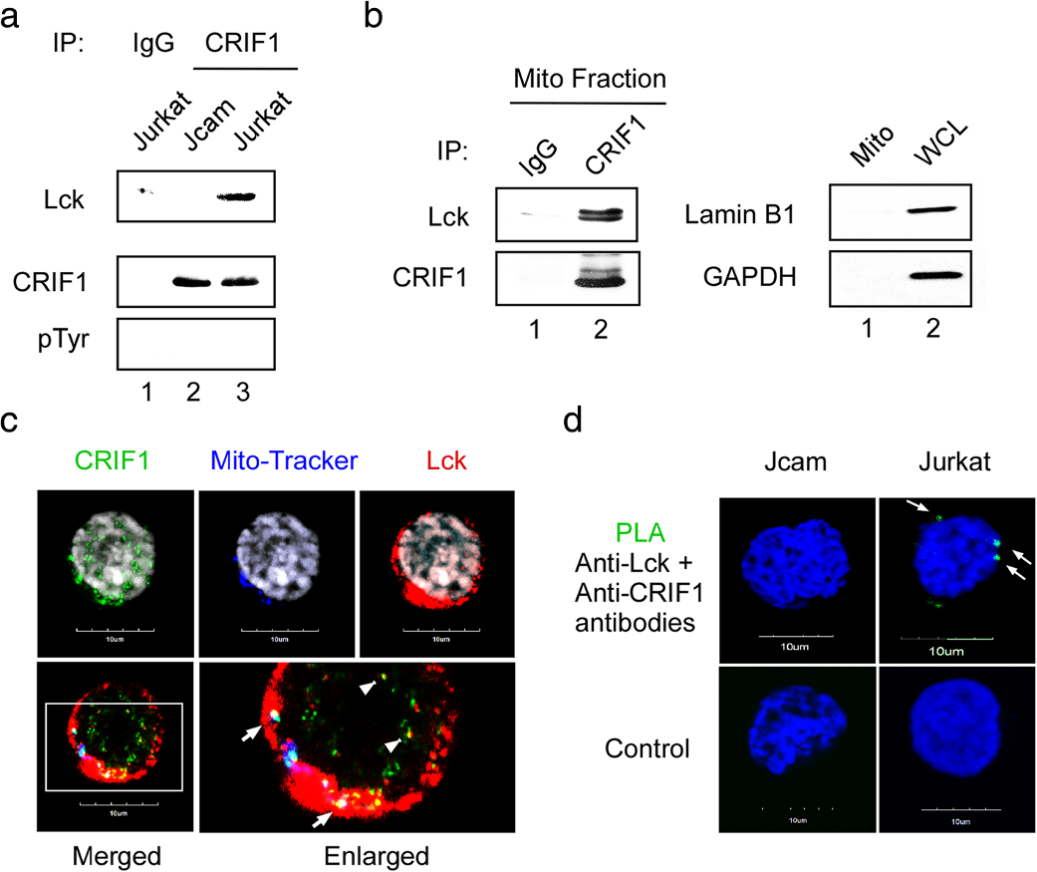
Lck interacts with mitochondrial CRIF1. (**a**) Jurkat and Jcam whole cell lysates were immunoprecipitated (IP) with anti-CRIF1 antibody, followed by Lck and CRIF1 immunoblotting. CRIF1 immunoblot was stripped and then reblotted with anti-phosphotyrosine (pTyr) antibody. Equal amounts of Jurkat whole cell lysate were also immunoprecipitated with normal IgG as a negative control (lane 1). (**b**) Mitochondrial proteins isolated from Jurkat cells were immunoprecipitated with either anti-CRIF1 antibody or control IgG, and then subjected to Lck and CRIF1 immunoblotting (left panels). A fraction of mitochondrial lysate was analyzed by lamin B1 and GAPDH immunoblotting to confirm the absence of nuclear and cytosolic contamination, respectively (right panels, lane 1). Jurkat whole cell lysate was used as a positive control (right panels, lane 2). (**c**) Jurkat cells were subjected to immunofluorescence microscopy with three-color staining for CRIF1 (green), mito-tracker (blue), and Lck (red). Cells were also stained with DAPI to visualize nuclei (grey on upper panels). An area of three-color merged image bordered with white lines is enlarged on the right to show better resolution (lower panels). White arrows indicate co-localization of Lck and CRIF1 in mitochondria (white dots). White arrowheads depict co-localization of Lck and CRIF1 in the nucleus (yellow dots). (**d**) Jurkat and Jcam cells were subjected to PLA microscopy using primary antibodies specific for Lck and CRIF1 (upper panels). Green fluorescence indicates Lck and CRIF1 interaction *in situ* (white arrows). Secondary antibodies alone were used as negative controls (lower panels). Scale bars of 10 μm are shown in the bottom of microscopy images

We first performed co-immunoprecipitation to confirm the finding from our proteomic analysis in LSTRA leukemia. Indeed, Lck was co-precipitated with CRIF1 in LSTRA cells (see Additional file 2). Similar interaction was also validated in human Jurkat leukemia expressing normal level of Lck kinase. We observed co-precipitation of Lck with CRIF1 in Jurkat, but not Jcam (Fig. 3a). As a protein tyrosine kinase, Lck has the potential to phosphorylate the associated CRIF1. However, CRIF1 proteins precipitated from both Jurkat and Jcam do not have detectable level of tyrosine phosphorylation (Fig. 3a, bottom panel). This is consistent with the lack of CRIF1 tyrosine phosphorylation reported in literature.

To evaluate Lck and CRIF1 interaction in the mitochondria, we performed co-immunoprecipitation using mitochondrial extracts prepared from Jurkat cells. As shown in Fig. 3b, Lck can be co-precipitated with CRIF1 in the mitochondrial fraction (left panels) free of nuclear and cytoplasmic contamination (right panels). To independently verify Lck-CRIF1 association in the mitochondria, we performed immunofluorescence microscopy. As shown in Fig. 3c, co-localization of CRIF1 and Lck can be detected in both mitochondrial (indicated by white arrows) and nuclear (indicated by white arrowheads) compartments of Jurkat cells.

To further confirm close-range interaction between Lck and CRIF1, we conducted *in situ* PLA microscopy. PLA relies on oligonucleotide hybridization and ligation when two target proteins are within 16 nm or below, which suggests true interaction. Subsequent amplification step and labeling with fluorophore gives a fluorescent dot at the exact site of protein interaction, which can be visualized by microscopy. As shown in Fig. 3d, PLA staining was observed outside the nucleus of Jurkat (upper-right panel). In contrast, no PLA staining was detected in Jcam that lacks Lck (upper-left panel). The specificity of PLA was further confirmed by the absence of fluorescent signal with secondary antibodies alone (Fig. 3d, lower panels). This is consistent with close interaction between Lck and CRIF1 in the mitochondria of Jurkat cells.

**Fig. 4:**
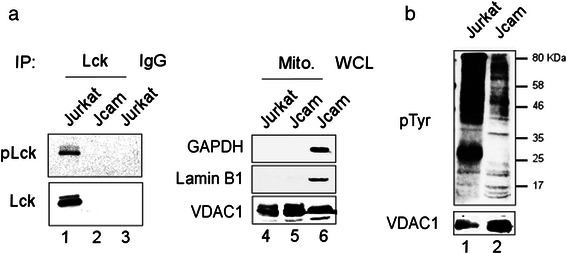
Active Lck kinase activity in Jurkat mitochondria. (**a**) Equal amount of proteins isolated from Jurkat and Jcam mitochondria were immunoprecipitated by anti-Lck (lanes 1 and 2) or control IgG (lane 3). Immunoprecipitates were blotted sequentially with antibodies specific for Tyr394-phosphorylated Lck (pLck) and total Lck (lanes 1-3). A small fraction of mitochondrial lysates were blotted for GAPDH, lamin B1 and VDAC1 to confirm fraction purity (lanes 4 and 5). Jcam whole cell lysate was included as a positive control for markers (lane 6). (**b**) Total proteins from mitochondrial fractions of Jurkat and Jcam cells were subjected to anti-phosphotyrosine immunoblotting (upper panel). Molecular weight markers are denoted on the right. VDAC1 immunoblot was used as a loading control (lower panel)

The absence of CRIF1 tyrosine phosphorylation in Jurkat cells (Fig. 3a) suggests that Lck interaction with CRIF1 may be independent of its kinase activity. To further determine whether mitochondrial Lck retains its kinase activity, we immunoprecipitated Lck from the mitochondrial fractions of Jurkat and Jcam cells (Fig. 4a, lanes 1 and 2). Lck kinase activity was confirmed by the phosphorylation of positive-regulatory Tyr394 in Jurkat, but not Jcam cells (upper panel). Consistent with the presence of active Lck kinase in Jurkat mitochondria, the overall level of mitochondrial protein tyrosine phosphorylation is significantly higher in Jurkat as compared to Jcam (Fig. 4b).

### Lck negatively regulates CRIF1-mediated translation of mitochondrion-encoded proteins

**Fig. 5:**
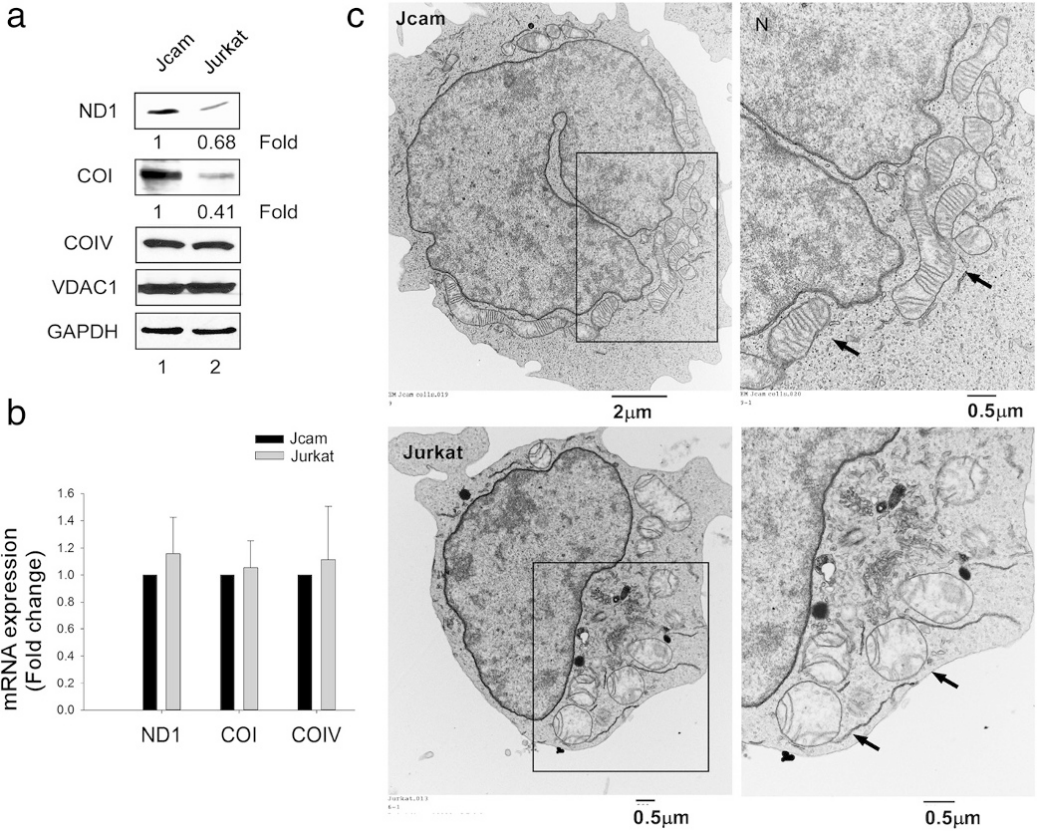
Lower levels of mitochondrion-encoded OXPHOS proteins and abnormal mitochondrial structure in Jurkat.( **a**) Normalized whole cell lysates from Jurkat and Jcam cells were analyzed by Western blot using antibodies specific for ND1, COI, COIV, VDAC1 and GAPDH. Signal intensity was quantitated for ND1 and COI and fold change is indicated below the images. (**b**) Total RNAs isolated from Jurkat and Jcam cells were subjected to real-time PCR using primers specific for human ND1, COI and COIV. Data from triplicates were normalized to actin and expressed as fold change of Jurkat in comparison to Jcam. Statistical analyses show no significant difference from three independent studies. (**c**) Transmission electron microscopy of Jurkat (lower panels) and Jcam (upper panels) cells. An area with enriched mitochondria is bordered with black lines and enlarged on the right to better visualize detailed intramitochondrial structure. Black arrows denote several representative mitochondria. The position of nucleus at the upper-left corner is also labeled as “N”. Scale bars are shown in the lower-right corners of microscopy images

CRIF1 is an essential component of translational machinery in the mitochondrion through its association with the mitoribosomes [12]. The observation of reduced mitochondrial respiration (Fig. 2) and mitochondrial Lck-CRIF1 interaction (Fig. 3) in Jurkat led us to hypothesize that Lck may be a negative regulator of mitochondrial CRIF1. We examined the levels of two ETC proteins encoded by the mitochondrial DNA: ND1 and COI. ND1 and COI are essential for the assembly of Complex I and Complex IV, respectively. The absence of these two core proteins leads to instability of the entire complexes. To determine the specificity of Lck’s inhibitory effect on ETC, we also analyzed the protein level of COIV, which is a component of Complex IV encoded by the nuclear genome. As shown in Fig. 5a, ND1 and COI protein levels are reduced in Jurkat as compared to Jcam cells. However, we detected no difference in COIV protein expression between Jurkat and Jcam cells. The nuclear-encoded mitochondrial outer membrane protein VDAC1 is also expressed at comparable levels (Fig. 5a). Lower levels of ND1 and COI protein expression are not the consequence of reduced mRNA level as shown by quantitative real-time PCR analysis (Fig. 5b). These results are consistent with the role of mitochondrial Lck as a negative regulator of CRIF1 in the translation of mitochondrion-encoded OXPHOS peptides.

In mouse cardiomyocytes, CRIF1 deficiency is known to cause abnormal mitochondrial structure with the loss of internal cristae and reduced mitochondrial respiration [50]. Similarly, our electron microscopy analysis showed Jurkat cells with bulged and swollen mitochondria (Fig. 5c, lower panels). The number of internal cristae from the folding of inner membrane in each mitochondrion is also greatly reduced with abnormal structure in Jurkat. This is in sharp contrast to the ellipse-shaped and elongated mitochondria with numerous cristae at a right angle from the outer membrane observed in Jcam cells (Fig. 5c, upper panels).

**Fig. 6:**
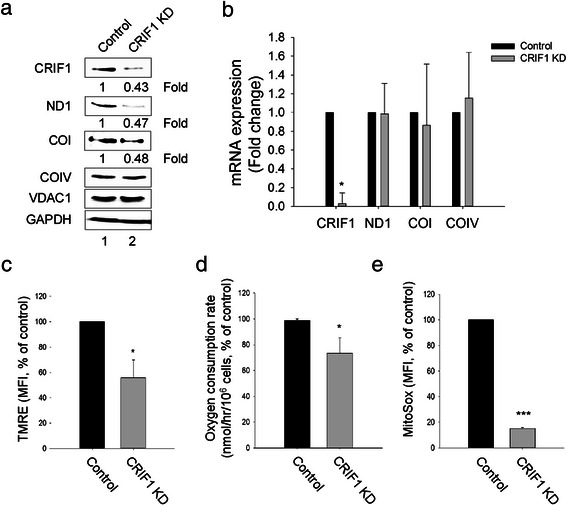
CRIF1 is required for normal expression of mitochondrion-encoded proteins. Comparisons are made between Jcam cells expressing scrambled shRNA (Control) and CRIF1-specific shRNA (CRIF1 KD). (**a**) Normalized whole cell lysates were subjected to immunoblotting for CRIF1, ND1, COI, COIV, VDAC1 and GAPDH. Signal intensity was quantitated for CRIF1, ND1 and COI and fold change is indicated below the images. (**b**) Total RNAs were subjected to real-time PCR using primers specific for human CRIF1, ND1, COI and COIV. Data from triplicates were normalized to actin and expressed as fold change of CRIF1 knock-down (CRIF1 KD) in comparison to control Jcam. (**c**, **d**, **e**) Mitochondrial membrane potentials, oxygen consumption rate, and mitochondrial superoxide levels were analyzed as described for Fig. 2. Data are presented as percentage of change from CRIF1 knock-down in comparison to control Jcam. Statistical analyses show the results from three independent studies, *p < 0.05, ***p < 0.001

To further explore whether these negative effects of Lck expression on mitochondria are due to its interaction with CRIF1, we knocked down CRIF1 in Jcam cells that lack mitochondrial Lck. The efficiency of CRIF1 knock-down was verified at both protein (Fig. 6a) and RNA (Fig. 6b) levels. Compared to control Jcam, Jcam cells with CRIF1 knock-down have reduced protein levels of ND1 and COI, but not COIV (Fig. 6a). Reduction of ND1 and COI protein expression is not due to lower levels of mRNA (Fig. 6b). These results show that CRIF1 removal has a similar effect as the presence of mitochondrial Lck in down-regulating the translational machinery within mitochondria. Consistent with reduced mitochondrial respiration in Jurkat cells (Fig. 2), CRIF1 silencing in Jcam cells also leads to lower levels of mitochondrial membrane potential (Fig. 6c), oxygen consumption (Fig. 6d), and mitochondrial superoxide (Fig. 6e). Consistent with our findings, CRIF1 deficiency in mouse adipose tissue also leads to reduced levels of mitochondrion-encoded OXPHOS proteins and subsequent decrease of mitochondrial respiration [51].

In summary, our data demonstrate reduced level of mitochondrion-encoded OXPHOS proteins and abnormal mitochondrial structure in Jurkat as compared to Jcam. Reduced synthesis of mitochondrion-encoded ETC components may lead to decreased mitochondrial respiration and altered mitochondrial morphology in Jurkat cells. Similar effects are observed when CRIF1 expression in Jcam cells is reduced by RNA silencing. These data support a crucial role of CRIF1 in intramitochondrial translational machinery and in maintaining ETC functions. They are also consistent with negative regulation of mitochondrial CRIF1 by Lck through direct interaction.

### Lck expression disrupts CRIF1 interaction with Tid1 protein

**Fig. 7:**
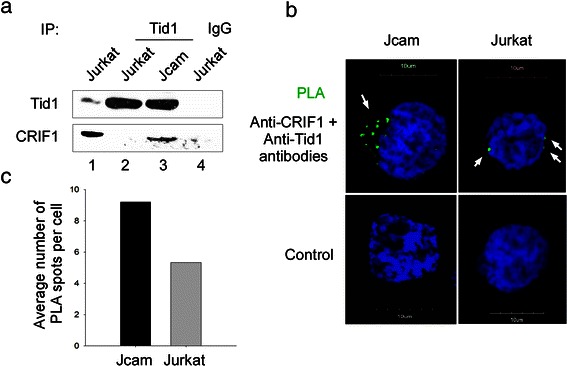
Decreased interaction of CRIF1 and Tid1 in Jurkat cells. (**a**) Whole cell lysates from Jurkat and Jcam cells were subjected to Tid1 immunoprecipitation and subsequent immunoblotting with anti-Tid1 and anti-CRIF1 antibodies. Jurkat lysate immunoprecipitated with normal IgG was used as a negative control (lane 4). A fraction of Jurkat lysate was loaded as a positive control (lane 1). (**b**) Jurkat and Jcam cells were analyzed by PLA microscopy using primary antibodies specific for CRIF1 and Tid1 (upper panels). Green fluorescence indicates CRIF1 and Tid1 interaction *in situ* (white arrows). Secondary antibodies alone were used as negative controls (lower panels). Scale bars of 10 μm are also shown. (**c**) The number of CRIF1 and Tid1 interacting complex were counted in multiple cells to obtain average number per cell

Through interaction with chaperon proteins, such as Tid1, mitochondrial CRIF1 is also important in proper folding and insertion of mitochondrion-encoded OXPHOS proteins into inner mitochondrial membrane [12]. We hypothesized that Lck interaction with CRIF1 in the mitochondria may interfere with CRIF1 and Tid1 association. To test this hypothesis, we compared interaction of CRIF1 with Tid1 in the presence and absence of Lck. Indeed, the amount of CRIF1 co-precipitated with Tid1 is greatly reduced in Jurkat as compared to Jcam (Fig. 7a, lower panel). PLA microscopy further confirms reduced association between CRIF1 and Tid1 in Lck-expressing Jurkat (Fig. 7b). Quantification of the PLA spots per cell indicates that, on average, there are more CRIF1-Tid1 complexes in Jcam (9.2 per cell) than in Jurkat (5.3 per cell) (Fig. 7c). These data suggest that Lck may competitively bind to CRIF1 and prevent proper folding and assembly of ETC complex in mitochondrial inner membrane. Combined with a reduction in protein expression (Fig. 5), they may coordinately contribute to lower mitochondrial respiration in Jurkat cells (Fig. 2).

## Discussion

**Fig. 8:**
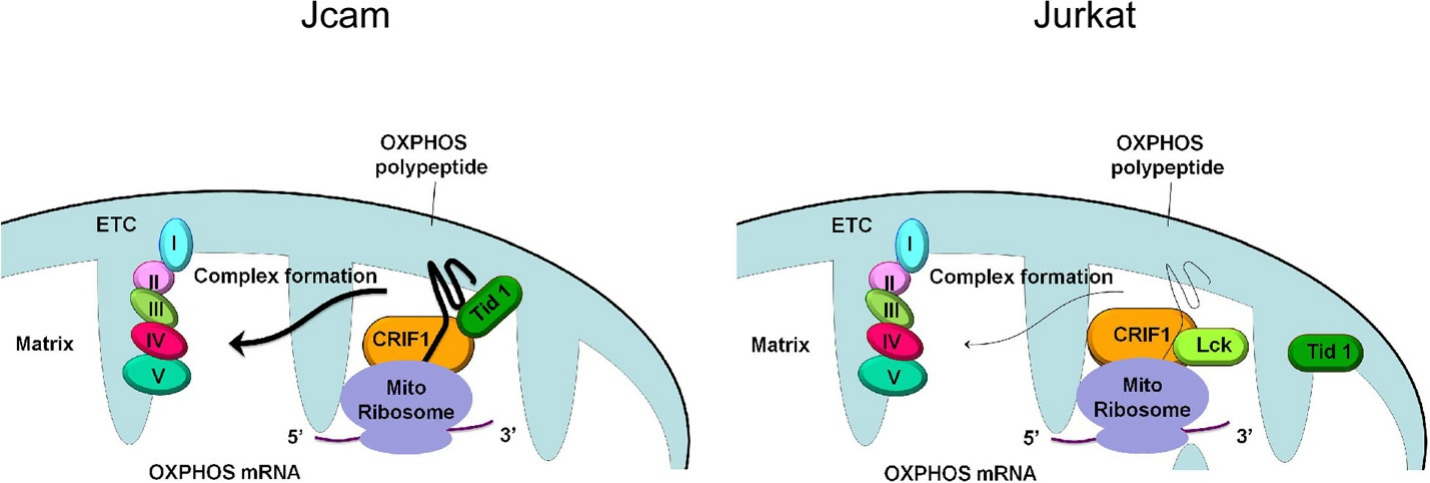
Schematic diagrams illustrating the potential mechanism of how Lck functions in mitochondria. As shown in Jcam, CRIF1 is a crucial component of the mitochondrial translational machinery and stabilizes the electron transport chain (ETC) complex. In Jurkat, Lck interaction with CRIF1 in mitochondria may disrupt CRIF1’s association with Tid1 and other translational components in the mitoribosome. Reduced levels of mitochondrion-encoded OXPHOS polypeptide and a defect in subsequent insertion into the inner membrane may lead to a decrease in mitochondrial respiration. The level of mitochondrion-encoded OXPHOS mRNA, however, remains unchanged in the mitochondrial matrix between Jurkat and Jcam cells

It has become increasingly evident that many cytoplasmic PTKs exhibit novel functions in other subcellular compartments [16, 21]. This non-canonical mode of signaling adds complexity to the paradigm of PTKs as cytoplasmic signal transducers in regulating cellular responses to extracellular stimuli. Consistent with the role of PTKs in oncogenesis, mitochondrial localization of PTKs has been specifically linked to metabolic shift, malignant progression, and resistance to chemotherapy in solid tumors [19, 20]. However, the role of mitochondrial PTKs in malignant transformation of hematopoietic cells remains poorly understood. In this study, we examined Jurkat and Jcam cells, the best characterized pair of human leukemic T-cell lines in studying Lck kinase. Our data demonstrate that Jurkat and Jcam cells have significant differences in mitochondrial OXPHOS (Fig. 2), which is accompanied by altered mitochondrial morphology (Fig. 5c). To the best of our knowledge, this is the first report that links Lck kinase with reduced mitochondrial respiration and confirms mitochondrial localization of Lck in blood cancer. Identification of CRIF1 as one of the Lck-interacting partners further reveals a novel mechanism of how PTK functions inside mitochondria (Fig. 8).

In contrast to previously reported roles of mitochondrial PTKs in phosphorylating various substrates within mitochondria, mitochondrial Lck does not phosphorylate the associated CRIF1 on tyrosine residues (Fig. 3a). Instead, our data suggest that mitochondrial Lck in Jurkat cells may compete with Tid1, a key component of the mitochondrial translational machinery, in binding to CRIF1 (Fig. 7). Disruption of the CRIF1-Tid1 interaction may lead to a defect in proper folding and insertion of newly synthesized OXPHOS polypeptides into mitochondrial inner membrane (Fig. 8). Subsequent interruption of ETC complex assembly can result in reduced mitochondrial respiration (Fig. 2). Knocking down CRIF1 expression in Jcam cells can disrupt the translational machinery in a similar manner to repress mitochondrial respiration (Fig. 6). The negative effect of mitochondrial Lck on OXPHOS can be further amplified by its inhibition on translation of mitochondrion-encoded mRNAs from mitoribosomes (Fig. 5a). It is possible that Lck may interfere with CRIF1 association with other components of mitoribosome through competitive binding. Mitochondrial Lck in Jurkat and CRIF1 silencing in Jcam may not affect the transcription of mitochondrial DNA because steady-state levels of mitochondrion-encoded mRNAs remain unchanged (Figs. 5b and 6b). This is the first example of regulating mitochondrial translational machinery by a PTK independent of its kinase activity.

It is important to note that we do not exclude the possibility that mitochondrial Lck may exert additional functions through tyrosine phosphorylation. Consistent with the presence of active mitochondrial Lck kinase, we did observe significantly higher levels of tyrosine phosphorylation in proteins isolated from Jurkat mitochondria as compared to Jcam mitochondria (Fig. 4). Our mass spectrometry analysis reveals other mitochondrial proteins that interact with Lck (Table 1). Adenine nucleotide translocator 1 (ANT1), for example, can be phosphorylated on Tyr194 by Lck upon stimulation to protect myocardial cells from apoptosis [52]. Interestingly, several previously reported PTK-targeted mitochondrial proteins, such as COII, pyruvate dehydrogenase kinase 1, and NADH dehydrogenase flavoprotein 2, are not identified as Lck-associated proteins in our mass spectrometry analysis of leukemic T-cells. It suggests that substrate specificity may exist for different PTKs. Alternatively, but not mutually exclusive, cell type specificity may also dictate mitochondrial localization of distinct PTK and the downstream effector proteins within mitochondria. Experiments are in progress to further validate these candidates identified by proteomics as the authentic Lck-interacting proteins.

Like many cytoplasmic PTKs, Lck protein does not have a discernible mitochondrial targeting sequence (MTS). Different mechanisms have been proposed for mitochondrial translocation of proteins that lack a conventional MTS. EGFR contains a non-conventional MTS in the juxtamembrane region to mediate translocation into mitochondria by endocytosis and retrograde movement upon stimulation [18]. Proteins without defined MTS often translocate into mitochondria by binding to other mitochondrial proteins with classical MTS. For example, ErbB2 mitochondrial localization depends on its association with mitochondrial heat shock protein 70 [20]. Tid1 also promotes the import of cytoplasmic p53 into mitochondria [53]. It is, therefore, possible that Lck may translocate into mitochondria through a similar mechanism of association with chaperone proteins. CRIF1 does have a distinct MTS at the very end of its amino-terminal region [12] and may aid in Lck translocation into mitochondria. The chaperone function may also be carried out by a yet-to-be identified non-conventional MTS within Lck or by other Lck-interacting proteins revealed by mass spectrometry (Table 1).

CRIF1 is a unique protein because, in addition to MTS, it also contains a classical nuclear localization signal. CRIF1 was originally identified as a nuclear protein that interacts and regulates the activity of several other nuclear proteins [47–49]. CRIF1 is also known as a tumor suppressor that inhibits cell proliferation and induces apoptosis in leukemic cells [47]. Considering the dual functions of CRIF1 in nuclear and mitochondrial compartments, we cannot rule out the possibility that CRIF1 silencing in Jcam cells may also affect some nuclear activity. Nevertheless, CRIF1 knock-down does not non-specifically alters nuclear gene expression, as shown by constant levels of nuclear-encoded COIV, VDAC1 and GAPDH (Fig. 6a). Consistent with previous report of nuclear Lck [38], CRIF1 and Lck interaction was also detected in the Jurkat nucleus (Fig. 3c). Similar to CRIF1 knock-down result, COIV, VDAC1 and GAPDH expression levels remain unchanged in Jurkat in comparison to Jcam (Fig. 5a). However, it is plausible that nuclear Lck may regulate other target genes that indirectly modulate mitochondrial activity. Experiments are in progress to specifically target wild-type and mutant Lck proteins into the mitochondria of Jcam cells and further elucidate their functional outcome.

Tumor cell’s dependence on glycolysis versus oxidative phosphorylation for ATP production can vary depending on cancer types and other factors [6–9]. A metabolic shift occurs when cancer cells use more glycolysis than oxidative phosphorylation to generate energy. The metabolic shift away from mitochondrial OXPHOS is a key step of this metabolic reprogramming. Our data of reduced mitochondrial OXPHOS in Jurkat cells support a similar metabolic shift in Lck-associated leukemia. During aerobic glycolysis in the cytoplasm, lactate is often produced by lactate dehydrogenase (LDH) through conversion of pyruvate, the end product of glycolysis. Interestingly, our proteomic analysis identified LDH as another potential Lck-interacting protein (Table 1). It suggests that cytosolic Lck may also regulate metabolic pathways in conjunction with reduced mitochondrial OXPHOS through mitochondrial Lck. These findings suggest an extensive crosstalk of nuclear, mitochondrial and cytosolic compartments in conferring cancer metabolism. The results from our current study on mitochondrial Lck represent an important piece of the puzzle to better understand how PTKs can be key players in coordinating this complex network.

## Conclusions

Our findings provide evidence that mitochondrial localization of Lck and its interaction with CRIF1 disrupt intramitochondrial translational machinery. Reduced levels of key ETC components may lead to abnormal mitochondrial structure with the loss of densely packed inner membrane. Subsequent repression of mitochondrial OXPHOS may contribute to metabolic shift toward aerobic glycolysis. These results represent a novel mode of Lck signaling in driving cancer metabolism in leukemia cells and, potentially, in other human cancer. Mechanistically, kinase-independent function of Lck is also distinct from all previously reported PTKs in mitochondria. Finally, mitochondria and energy metabolism has become increasingly important as targets in cancer therapy [54]. Our work has identified potentially new molecular targets for future development of therapeutic strategies in redirecting metabolic pathways of human cancer.

## Additional files

Additional file 1: Figure S1. Oxygen consumption rates were measured in Jurkat and Jcam cells both before (Basal) and after adding oligomycin. Oxygen consumption rate in Jcam without oligomycin was set as 100 % for comparison. Statistical analyses show the result from three independent experiments, **p*<0.05, ***p*<0.01.
Additional file 2: Figure S2. Lck and CRIF1 interaction in mouse LSTRA leukemia. Equal amount of total proteins from LSTRA whole cell lysate were immunoprecipitated with anti-CRIF1 antibody or control IgG. Immunoprecipitates were then subjected to Lck (upper panel) and CRIF1 (lower panel) immunoblotting.

## Abbreviations

OXPHOS: Oxidative phosphorylation
ETC: Electron transport chain
CRIF1: CR6-interacting factor 1
Tid1: Tumorous imaginal disc 1
PTK: Protein tyrosine kinase
EGFR: Epidermal growth factor receptor
FGFR1: Fibroblast growth factor receptor 1
COII: Cytochrome *c* oxidase subunit II
SFK: Src family kinase
Lck: Lymphocyte-specific protein tyrosine kinase
TCR: T-cell receptor
PBS: Phosphate-buffered saline
VDAC1: Voltage-dependent anion channel 1
ND1: NADH dehydrogenase subunit 1
COI: Cytochrome *c* oxidase subunit I
COIV: Cytochrome *c* oxidase subunit IV
GAPDH: Glyceraldehyde 3-phosphate dehydrogenase
PLA: Proximity ligation assay
SPB: Sorensen’s sodium phosphate buffer
TMRE: Tetramethylrhodamine, ethyl ester
ROS: Reactive oxygen species
ANT1: Adenine nucleotide translocator 1
MTS: Mitochondrial targeting sequence
LDH: Lactate dehydrogenase
