# Supplementary figures and images for: Lymphocyte-specific protein tyrosine kinase (Lck) interacts with CR6-interacting factor 1 (CRIF1) in mitochondria to repress oxidative phosphorylation
Source: BMC Cancer. 2015 Jul 26;15:551. doi: 10.1186/s12885-015-1520-6 (PMC4515320; doi:10.1186/s12885-015-1520-6)

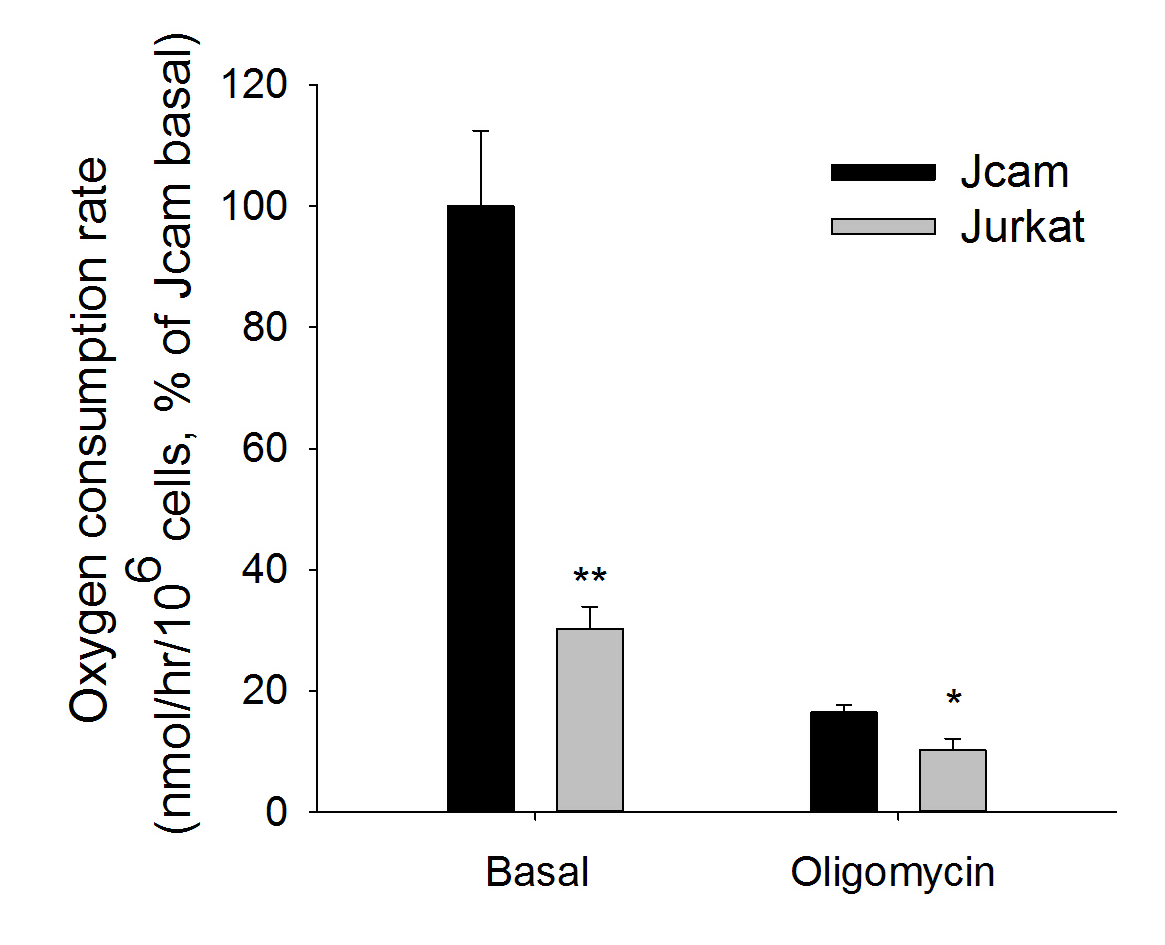

Supplement: Additional file 1: Figure S1. — Oxygen consumption rates were measured in Jurkat and Jcam cells both before (Basal) and after adding oligomycin. Oxygen consumption rate in Jcam without oligomycin was set as 100 % for comparison. Statistical analyses show the result from three independent experiments, *p<0.05, **p<0.01. [file 12885_2015_1520_MOESM1_ESM.tiff]

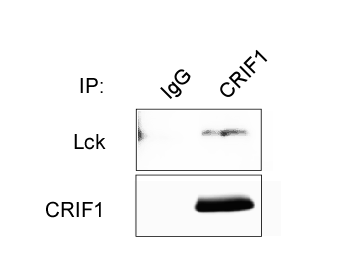

Supplement: Additional file 2: Figure S2. — Lck and CRIF1 interaction in mouse LSTRA leukemia. Equal amount of total proteins from LSTRA whole cell lysate were immunoprecipitated with anti-CRIF1 antibody or control IgG. Immunoprecipitates were then subjected to Lck (upper panel) and CRIF1 (lower panel) immunoblotting. [file 12885_2015_1520_MOESM2_ESM.tiff]
